# When Bleeding Is Only the Beginning: A Rare Complication Following Hemorrhage From a Meckel’s Diverticulum

**DOI:** 10.7759/cureus.95605

**Published:** 2025-10-28

**Authors:** Elena Kkoumourou, Maria Florou, Vassileios Lambropoulos, Maria Tsopozidi, Vassileios Mouravas, Christos Kaselas

**Affiliations:** 1 2nd Department of Pediatric Surgery, Aristotle University of Thessaloniki, Papageorgiou General Hospital, Thessaloniki, GRC; 2 1st Department of Pediatric Surgery, Aristotle University of Thessaloniki, Gennimatas General Hospital of Thessaloniki, Thessaloniki, GRC

**Keywords:** colectomy, gastrointestinal hemorrhage, ileostomy, ischemic colitis, meckel’s diverticulum, pediatric surgery

## Abstract

Meckel’s diverticulum is the most common congenital anomaly of the gastrointestinal tract and may present with complications such as bleeding, obstruction, or inflammation. Massive lower gastrointestinal bleeding in adolescents is rare and may be life-threatening. Ischemic colitis is extremely uncommon in this age group and has not been previously described as a complication of gastrointestinal hemorrhage, following resection of a bleeding Meckel’s diverticulum. We report a case of an adolescent boy who presented with persistent ileus due to ischemic colitis, following extensive hemorrhage of the Meckel’s diverticulum.

## Introduction

Meckel’s diverticulum is the most common congenital anomaly of the gastrointestinal tract, resulting from incomplete obliteration of the omphalomesenteric duct during the fifth week of gestation [1,2]. It is a true diverticulum, which means it contains all layers of the bowel wall. It is commonly found 7-200 cm from the ileocecal valve on the antimesenteric margin of the ileum and occurs in approximately 2% of the population [3]. Generally, in most cases, it is asymptomatic, but complications such as obstruction, diverticulitis, bleeding, and perforation may appear, more commonly in the pediatric population [4]. The most common presenting symptom in children and adolescents is painless gastrointestinal bleeding, often caused by ulceration from ectopic gastric mucosa within the diverticulum [4,5]. The current patient, after extensive Meckel’s bleeding, had a complicated postoperative period with recurrent ileus manifestations and findings suggestive of ischemic colitis. The pathophysiology pathway of this clinical entity starts with excessive bleeding and continues with hypovolemia and the subsequent hypoperfusion. At the same time, the vulnerable portions of the colon are susceptible to ischemia in the context of global hypoperfusion [4-6]. Early recognition of symptomatic Meckel’s diverticulum and its complications, followed by surgical resection, is generally curative, with low rates of morbidity. To the best of our knowledge, this is the first reported case of ischemic colitis in an adolescent boy, following massive bleeding due to symptomatic Meckel’s diverticulum.

## Case presentation

A 16-year-old male patient, previously healthy with a background of intense physical activity, presented to the emergency department with an episode of painless rectal bleeding associated with syncope. He reported two prior episodes of hematochezia over the preceding 48 hours. Notably, he had sought medical attention at a different on-call hospital two days earlier for similar painless rectal bleeding. At that time, he was discharged with a presumptive diagnosis of an anal fistula and instructions to return if symptoms recurred.

On arrival, he was pale and hypotensive, with a blood pressure of 89/60 mmHg and a heart rate of 147 bpm. The physical examination was otherwise unremarkable, except for the presence of blood on digital rectal examination. Immediate resuscitation was initiated, along with continuous monitoring and blood sampling for laboratory tests. The initial hemoglobin level was 6.5 g/dL (normal range: 12-15.4 g/dL) with a hematocrit level of 25% (normal range: 37%-46%). Aggressive intravenous hydration with crystalloid fluids and two units of packed red blood cells was commenced, and vital signs were continuously monitored. Due to the combination of hemodynamic instability and high clinical suspicion for a bleeding Meckel’s diverticulum, the decision was made to proceed with emergency exploratory laparotomy.

**Figure 1:**
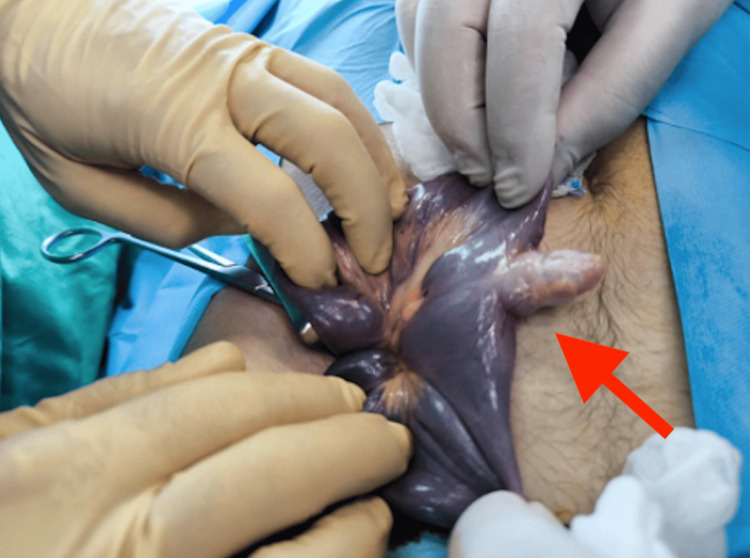
A bleeding Meckel’s diverticulum was identified 30 cm proximal to the ileocecal valve (arrow). The small bowel was noted to contain hemorrhagic material throughout its length.

Intraoperatively, a bleeding Meckel’s diverticulum was identified 30 cm proximal to the ileocecal valve and managed with a segmental ileal resection including the base of the diverticulum, followed by an end-to-end anastomosis. The small bowel was noted to contain hemorrhagic material throughout its length (Figure 1).

**Figure 2:**
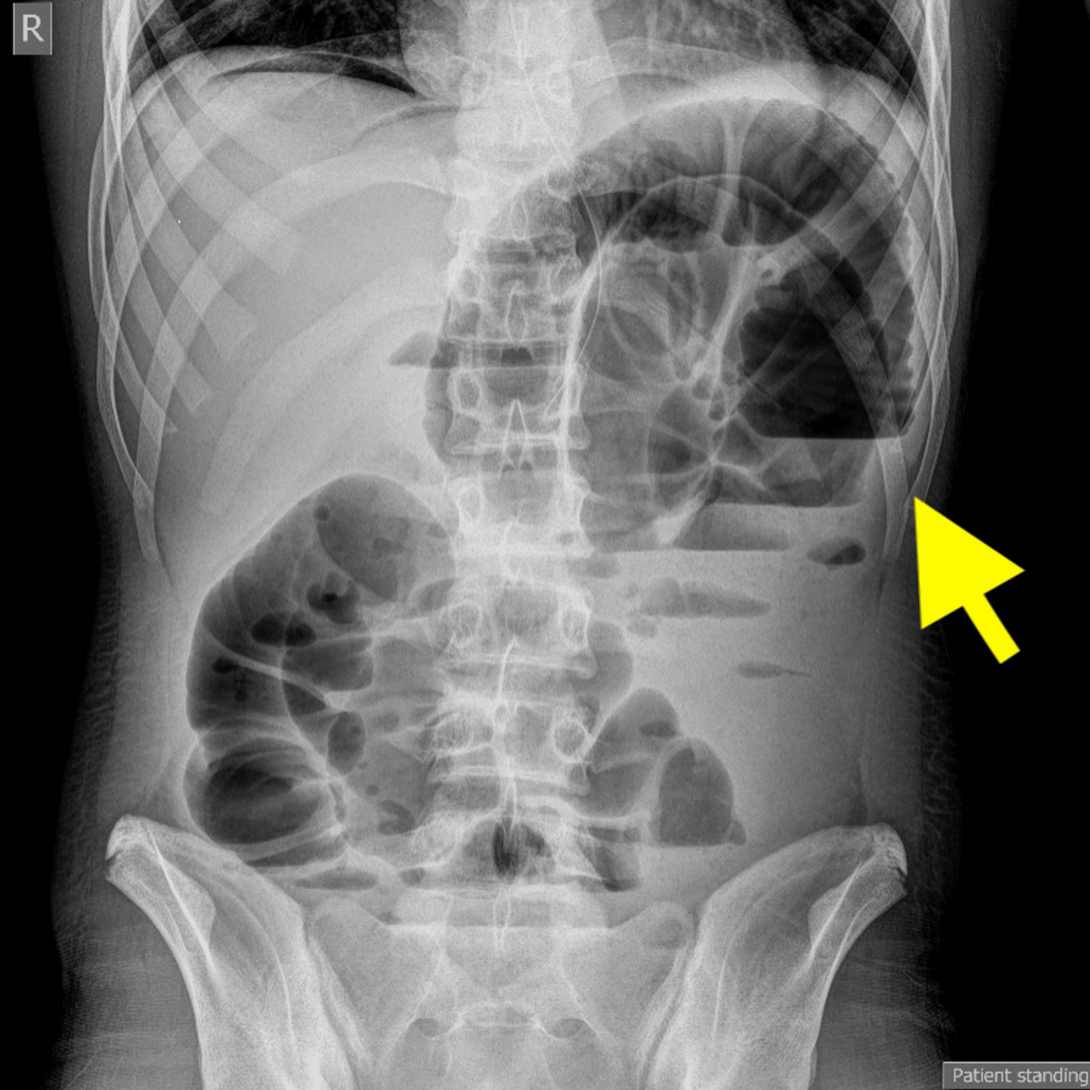
Presence of ileus in abdominal X-ray image. The arrow points to the abdominal distension (POD 5). POD: postoperative day

The patient was transferred to the surgical ward after the operation. The postoperative hemoglobin level was 9 g/dL with a hematocrit level of 27%. Despite an initially uneventful postoperative course, on postoperative day (POD) 5, he developed abdominal distension, high-pitched bowel sounds, and abdominal pain. Continuous imaging studies (abdominal X-ray) confirmed the presence of ileus (Figure 2).

His clinical condition remained unchanged despite conservative management. On postoperative day 8, the patient was taken back to the operating room, where a second exploratory laparotomy and adhesiolysis were performed. Intraoperatively, the anastomosis was inspected and found to be intact and patent. The patient returned to the surgical ward with a nasogastric tube in place, which drained increased volumes. Postoperatively, the abdominal distension persisted, accompanied by high-pitched bowel sounds and intermittent abdominal pain. Imaging studies continued to demonstrate features consistent with postoperative ileus, and the nasogastric tube yielded several liters of bilious fluid per day. Despite conservative management, the patient did not improve and was taken back to the operating room nine days after the second surgery (and 17 days after the first operation), with a diagnosis of adhesive bowel obstruction. During the third exploratory laparotomy, adhesiolysis was performed, and a loop ileostomy was created. The patient returned to the pediatric surgical ward with a nasogastric tube in place and was started on total parenteral nutrition (TPN) via a central venous catheter. This time, his postoperative course was truly uneventful. From the early postoperative days, the ileostomy began producing gas and watery output. During his hospitalization, the patient was mobilized, remained afebrile, and maintained adequate urine output. In the primary postoperative period, the nasogastric tube produced significant output, which gradually decreased after postoperative day 5. A few days later, the nasogastric tube was removed, and oral fluid intake was initiated. Feeding was well tolerated, and on clinical examination, the abdomen remained soft, non-tender, and compressible.

During the first 72 hours of oral feeding, the ileostomy output was high and watery, and reached volumes of 3-3.5 liters per day. A pediatric gastroenterology consultation was requested to assist with nutritional management and optimization of fluid and electrolyte balance. Gradually, the ileostomy output decreased in volume and became more feculent in consistency. Oral intake was progressively advanced, and by postoperative day 13 (and hospitalization day 21), the patient was tolerating a complete oral diet without complications. As part of the differential diagnosis, investigations were performed for inflammatory bowel disease, systemic lupus erythematosus, and vasculitis. Considering the day of acute bleeding and the segmental ileal resection (first operation) as day 0, we shortly present a timeline of the patient: POD 5-7: ileus, POD 8: second laparotomy and adhesiolysis, POD 17: third laparotomy, colectomy, and loop ileostomy, POD 24: start of oral feeding, and POD 31: discharge. Throughout the patient’s hospitalization, coagulation parameters remained within normal limits. After a month of hospitalization and three operations, the boy was discharged home, with a hemoglobin level of 12.3 g/dL, hematocrit level at 35%, and a normal biochemical panel.

**Figure 3:**
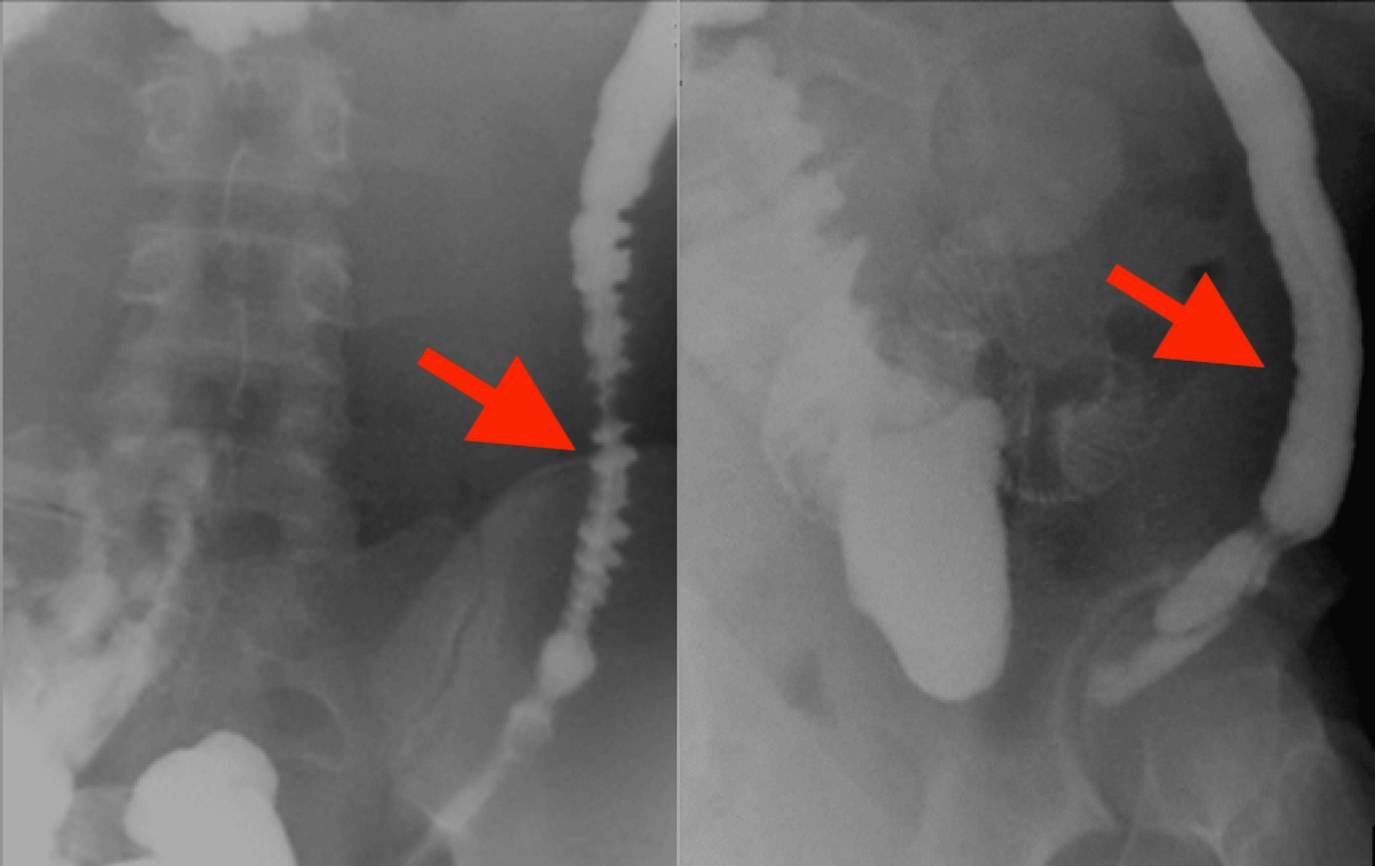
Contrast enema via the rectum: The descending colon (arrows) appeared hypoplastic, with markedly reduced caliber, diminished peristalsis, and loss of normal haustration, findings consistent with a “microcolon”-like appearance.

Three months later, prior to ileostomy closure, the patient underwent preoperative imaging to evaluate distal bowel integrity. A contrast enema was performed via the rectum, which demonstrated prompt progression of contrast through the rectum and proximal colon. However, the descending colon appeared hypoplastic, with markedly reduced caliber, diminished peristalsis, and loss of normal haustration, findings consistent with a “microcolon”-like appearance (Figure 3).

**Figure 4:**
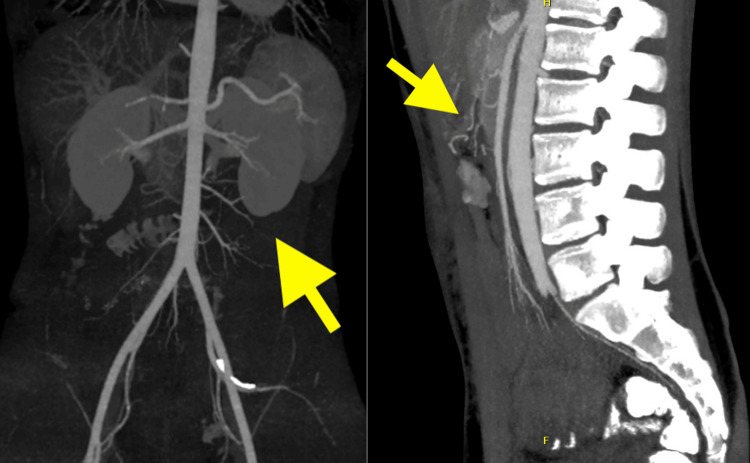
CT angiography revealed a delicate left colic artery branching toward the splenic flexure. CT: computed tomography

Subsequent computed tomography (CT) angiography revealed a delicate left colic artery branching toward the splenic flexure. Imaging confirmed segmental luminal narrowing and contraction of the descending colon without evidence of mechanical obstruction. The colonic wall demonstrated absent contrast enhancement and complete loss of its regular haustral pattern, consistent with segmental ischemia. Based on these radiologic findings, a diagnosis of ischemic colitis was suspected (Figure 4).

**Figure 5:**
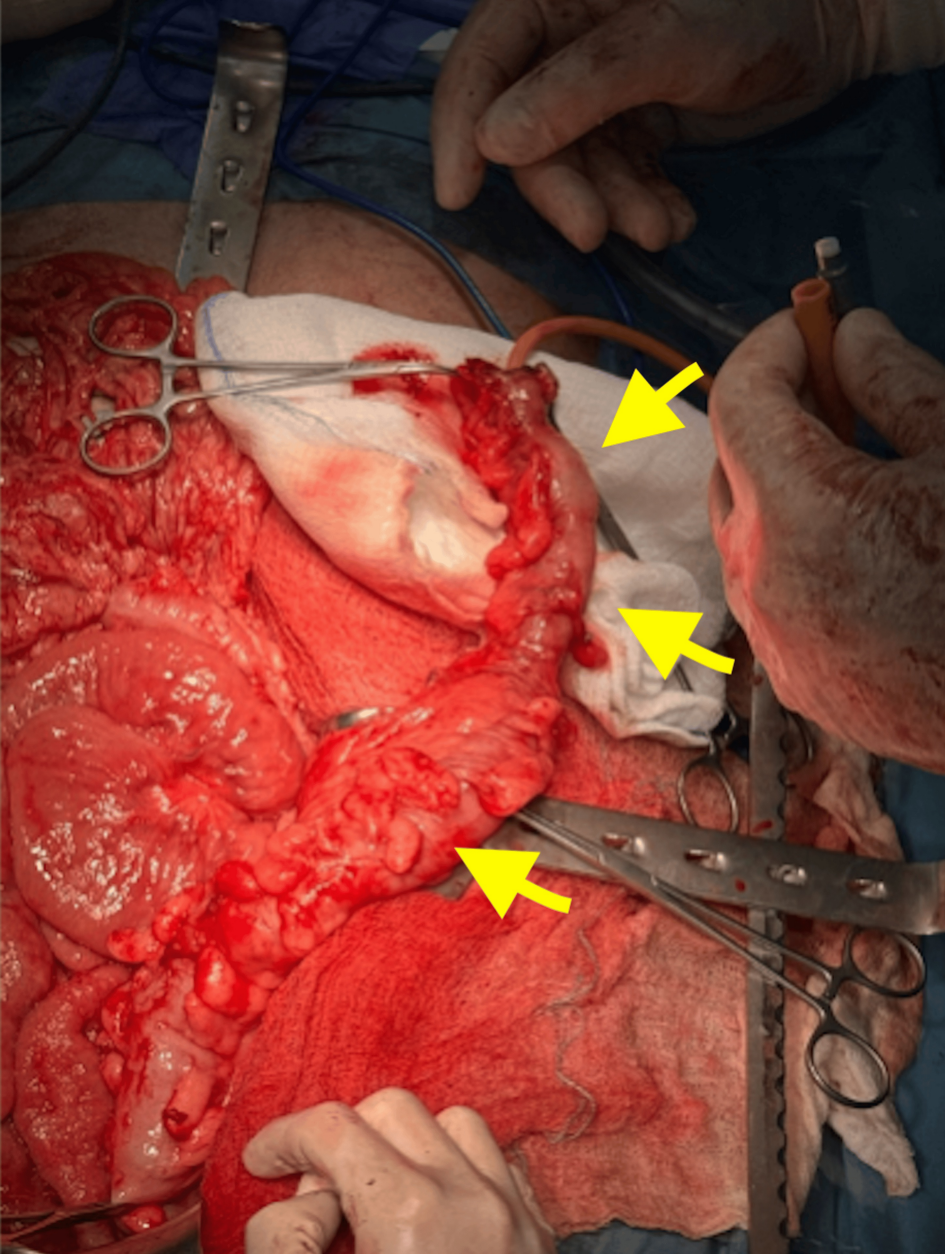
The resected colonic segment was examined by introducing a Foley catheter into its lumen. The figure demonstrates the difference in caliber between the normal (lower arrow) and diseased segment (upper and middle arrow), thickening of the omental appendages, and complete loss of the regular haustral pattern.

After multidisciplinary discussion, the patient underwent a fourth surgical procedure: an extended left hemicolectomy with primary end-to-end anastomosis and simultaneous closure of the loop ileostomy. Intraoperatively, the colonic segment affected by colitis was macroscopically identified, extending from the splenic flexure through the entire descending colon and into the proximal third of the sigmoid colon. The diseased segment was resected using a stapling device. A formal extended left hemicolectomy was completed, and the specimen was sent for histopathological examination (Figure 5). The results of the histopathological examination confirmed the diagnosis of ischemic colitis, as there were mucosal necrosis, congestion, and bleeding between the layers of the resected specimen.

The procedure was completed uneventfully. The postoperative course was uncomplicated. The patient resumed oral intake gradually and tolerated feeding well. Bowel function returned promptly, and he was discharged in good general condition. At follow-up, he remained asymptomatic, with regular bowel habits and no recurrence of symptoms.

## Discussion

Meckel’s diverticulum is the most common congenital anomaly of the gastrointestinal tract and affects about 2% of the population. Although it remains asymptomatic in most cases, it may present with complications such as bleeding, inflammation, obstruction, or perforation, especially in pediatric patients. Among these, painless lower gastrointestinal bleeding is the most expected presentation in children, usually due to ulceration caused by ectopic gastric mucosa within the diverticulum [2]. Surgical resection remains the treatment of choice in cases of symptomatic or bleeding Meckel’s diverticulum. Segmental small bowel resection, as performed in this case, is often preferred over wedge excision in order to ensure complete removal of possible ectopic tissue and minimize the risk of recurrence [5]. The patient described here underwent an urgent enterectomy due to hemodynamic instability and active bleeding, followed by primary anastomosis. Due to persistent ileus, during the short-term postoperative period, the patient had two additional exploratory laparotomies, which eventually led to the formation of a temporary loop ileostomy. Although postoperative ileus is not uncommon in pediatric patients undergoing abdominal surgery, the severity and duration observed in this case were unusual. Moreover, the development of ischemic colitis in an otherwise healthy adolescent represents an exceptional complication that has not been previously described in the context of Meckel’s diverticulum surgery, to our knowledge.

Several pathophysiological mechanisms may explain the presence of ischemic colitis in this patient: First of all, profound hypovolemia due to acute gastrointestinal bleeding may have led to transient mesenteric hypoperfusion, particularly in the watershed regions of the colon, such as the splenic flexure and descending colon [6]. The colon is more prone to ischemia compared with the small intestine because it receives a lower blood supply and its microvascular network is less developed. Blood supply to the colon is primarily provided by two major vessels: the superior mesenteric artery, which supplies the right colon and the transverse colon, and the inferior mesenteric artery, which supplies the left colon and sigmoid colon. The rectum receives blood from branches of the internal iliac arteries. Collateral circulation between the superior and inferior mesenteric systems provides some protection against ischemic injury. However, this network can differ between individuals. Therefore, the so-called “watershed zones” are areas where two arterial territories meet, such as the splenic flexure and the sigmoid colon, and are limited in collateralization, making them susceptible to ischemia [7]. Furthermore, there is emerging evidence that competitive athletics or a history of intense training may act as a predisposing factor for the development of ischemic events. A persistent, vigorous exercise can reduce splanchnic blood flow by 60%-80% due to sympathetic shunting of blood toward active muscles, thereby initiating transient intestinal hypoperfusion in susceptible individuals. This physiological stress, when repeated over time, may predispose to chronic ischemic changes in the colon that could potentially escalate, under extreme circumstances, into ischemic colitis [8].

Ischemic colitis is extremely rare in adolescents. In the pediatric population, reported causes include vasculitis, coagulopathies, and abdominal trauma, but very few cases have been documented following hemorrhage in otherwise healthy individuals [9,10]. In the current patient, the diagnosis was established with a contrast enema and a CT angiography, which detected a focal stricture and an impaired perfusion in the descending colon, respectively. Later, the diagnosis was confirmed macroscopically during surgery, as well as by histopathological examination. The need for definitive management of the ischemic segment and restoration of bowel continuity guided the decision to perform a left hemicolectomy with ileostomy closure.

## Conclusions

This is one of the very few reports discussing the occurrence of ischemic colitis in a teenage boy, following extensive bleeding of the Meckel’s diverticulum. In the postoperative period, especially when the recovery diverges from the expected course, it is important to maintain a comprehensive differential diagnosis and prompt treatment. Early identification of complications, such as persistent ileus and segmental ischemia, is crucial for timely intervention.
